# The Role of Astrocyte Dysfunction in Parkinson’s Disease Pathogenesis

**DOI:** 10.1016/j.tins.2017.04.001

**Published:** 2017-06

**Authors:** Heather D.E. Booth, Warren D. Hirst, Richard Wade-Martins

**Affiliations:** 1 Oxford Parkinson’s Disease Centre, University of Oxford, Oxford, UK; 2 Department of Physiology, Anatomy and Genetics, University of Oxford, Oxford, UK; 3 Biogen, Cambridge, MA, USA

## Abstract

Astrocytes are the most populous glial subtype and are critical for brain function. Despite this, historically there have been few studies into the role that they may have in neurodegenerative diseases, such as Parkinson’s disease (PD). Recently, however, several studies have determined that genes known to have a causative role in the development of PD are expressed in astrocytes and have important roles in astrocyte function. Here, we review these recent developments and discuss their impact on our understanding of the pathophysiology of PD, and the implications that this might have for its treatment.

## Trends

PD is characterised by the degeneration of dopaminergic neurons in the substantia nigra pars compacta (SNc).

Neuroinflammation in the SNc is a consistent feature of PD.

Recent studies of eight proteins implicated in PD demonstrate that they have functional roles in astrocyte biology.

These studies provide evidence that PD may develop, at least in part, due to astrocyte dysfunction.

## Astrocytes in Parkinson’s disease

Glia account for over 50% of the cells in the brain and can be divided into various subtypes, of which astrocytes are the most populous 1, 2. Although the existence of astrocytes was first documented over 100 years ago, relatively few studies are conducted into their roles in neurological disorders and diseases. PD is a common neurodegenerative disease, pathologically characterised by the loss of **dopaminergic neurons** (see Glossary) in the **substantia nigra pars compacta** (SNc) [3]. The mechanisms of this neuronal degeneration have not yet been clearly elucidated, although astrocytes have been implicated in the pathogenesis of PD. A key aspect of PD pathophysiology is neuroinflammation in the SNc, including the presence of reactive astrocytes 4, 5. This neuroinflammation has long been considered a downstream response to the death of dopaminergic neurons. However, evidence is building to suggest that astrocytes have an initiating role in PD pathophysiology.

Astrocytes have a range of functions, many of which are essential for maintaining neuronal health. They provide structural and metabolic support, and regulate synaptic transmission, water transport, and blood flow within the brain [6]. They produce various neurotrophic molecules, including glial-derived neurotrophic factor (GDNF), which is especially important for the development and survival of dopaminergic neurons 7, 8. Astrocytes also contribute to the blood–brain barrier, which has been shown to be disrupted in patients with PD [9]. Additionally, when an immune response is initiated by microglia, astrocytes surround the area, creating a barrier to prevent the spread of toxic signals into the surrounding healthy tissue [6].

**Figure 1:**
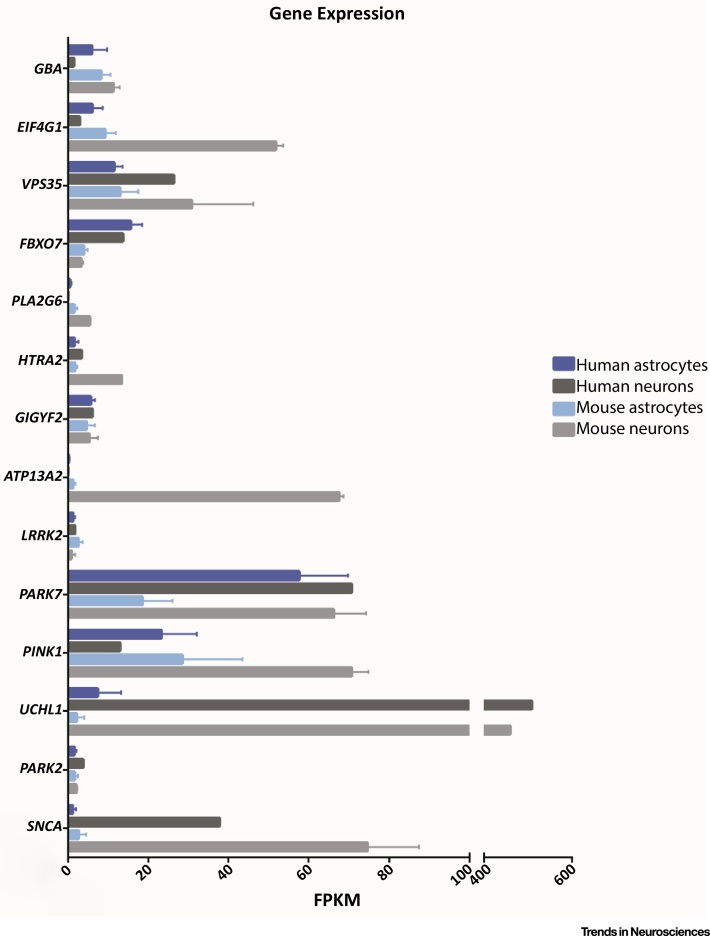
Expression Levels of Key Parkinson’s Disease (PD) Genes in Astrocytes and Neurons. Transcriptome data from Zhang *et al.*[11] showing the expression levels of genes known to be causative in PD in astrocytes and neurons from humans and mice. Human astrocytes *N* = 12 subjects; human neurons *N* = 1 subject; mouse astrocytes *N* = 6 animals; mouse neurons *N* = 2 animals. Abbreviation: FPKM, fragments per kilobase of transcript per million mapped reads. Graph shows mean ± SD. Data obtained from Supplementary Table S4 in the original publication [11], and can be browsed online at http://web.stanford.edu/group/barres_lab/brainseq2/brainseq2.html. For full gene names, please see the main text.

Evidence is emerging to suggest that disruption of astrocyte biology is involved in dopaminergic neuron degeneration in PD. Although most PD cases are idiopathic, monogenic mutations in 17 genes have been identified and implicated in the development of the disease [10]. A recent study comparing the transcriptome of different human and mouse brain cell subtypes demonstrated that many of the genes where monogenic mutations have been identified are expressed in astrocytes at levels comparable to, or in some cases higher than in, neurons (Figure 1) [11]. So far, proteins encoded by eight of these genes have been shown to have a role in astrocyte biology (Table 1). Here, we review these new findings and discuss their implications in the development of the disease.

**Table 1:** Genes That Are Causative in the Development of PD, and the Aspects of Astrocyte Biology in Which They Have Been Implicated

Gene	Protein	Astrocyte function	Refs
*PARK7*	DJ-1	Glutamate uptake	[15]
		Inflammatory response	14, 20, 21, 22
		Mitochondrial function	25, 26, 27
		Neurotrophic capacity	23, 24, 25, 26
		Oxidative stress	25, 26
*SNCA*	α-synuclein	Endocytosis	35, 36, 37, 38
		Fatty acid metabolism	[31]
		Glutamate uptake	[40]
		Inflammatory response	37, 38, 39
		Neurotrophic capacity	[40]
		Water transport	[40]
*PLA2G6*	Group VI Ca^2+^-independent phospholipase A_2_ (iPLA_2_)	Calcium signalling	43, 44
		Fatty acid metabolism	[42]
		Inflammatory response	[44]
*ATP13A2*	Lysosomal type 5 ATPase (ATP13A2)	Inflammatory response	[49]
		Lysosome function	[49]
		Neurotrophic capacity	[49]
*LRRK2*	Leucine-rich repeat kinase 2 (LRRK2)	Autophagy	[55]
		Lysosome function	[56]
*GBA*	β-Glucocerebrosidase (GCase)	Autophagy	[61]
		Lysosome function	[62]
		Mitochondrial function	[62]
*PINK1*	PTEN-induced putative kinase 1 (PINK1)	Embryonic development	[64]
		Mitochondrial function	[65]
		Proliferation	64, 65
*PARK2*	Parkin	Inflammatory response	[73]
		Mitochondrial function	[71]
		Neuroprotection	[69]
		Proliferation	69, 70
		Unfolded protein response	[72]

## Parkinson’s Disease-Related Genes and Their Functional Roles in Astrocytes

### DJ-1

The most evidence for a PD-related gene having a role in astrocyte biology has been found for DJ-1, which is encoded by the *PARK7* gene. Expression of *PARK7* has been shown to be higher in astrocytes than in neurons in postmortem human brain samples, and to be upregulated in reactive astrocytes in patients with PD 12, 13. Recent studies found that DJ-1 associates with, and regulates assembly of, lipid rafts in astrocytes 14, 15. Lipid rafts are highly organised membrane microdomains that are involved in membrane receptor trafficking, endocytosis, and signal transduction [16]. They are most commonly found in the plasma membrane, although they have also been reported in the membranes of cellular organelles [17]. Mutations in *Park7* were shown to result in the increased degradation of the lipid raft proteins flotillin-1 and caveolin-1, and to lead to disrupted lipid raft assembly [15]. As a result of this disruption, *Park7* knockout (KO) and mutant astrocytes were found to exhibit impaired glutamate uptake [15]. This effect was shown to be due to a decrease in protein expression of EAAT2, an astrocyte-specific glutamate transporter that has previously been shown to assemble in lipid rafts [18]. This reduction in glutamate uptake may result in high levels of glutamate in the extracellular space, leading to neuronal excitotoxicity, a known cause of neurodegeneration [19].

Lipid rafts also have an important role in astrocyte immune response to lipopolysaccharide (LPS). It has been shown that the disruption of lipid raft assembly that occurs in the absence of DJ-1 results in impaired TLR3/4-mediated endocytosis [14]. It has also been shown that *Park7*-KO astrocytes display alterations in inflammatory cytokine production 14, 20, 21, which could indicate a failure to terminate TLR4 surface signalling 14, 20. The role of DJ-1 in astrocyte immune function has been further investigated by treating *Park7*-KO astrocytes with IFN-γ [22]. It was found that DJ-1 regulates astrocyte inflammatory response to IFN-γ by facilitating the formation of a complex of p-STAT1 with its phosphatase SHP1, leading to dephosphorylation of p-STAT1 and termination of signalling. This process was shown to be neuroprotective; IFN-γ treatment resulted in increased neuronal toxicity in *Park7*-KO brain slices compared with wild-type (WT).

Knockdown or KO of *Park7* in astrocytes also results in a reduction in their ability to protect neurons against neurotoxicity in rotenone and 6-hydroxydopamine neurotoxin models of PD 23, 24. Additionally, overexpression of WT *Park7* increases the neuroprotective capacity of astrocytes in the rotenone model [23]. Further studies into the mechanism of neuroprotection in these models have suggested that DJ-1 has a role in mitochondrial function and oxidative stress. One study tested the neuroprotective effects of DJ-1 in the presence of inhibitors of different parts of the mitochondrial electron transport chain and found that its effects were specific to mitochondrial complex I [25]. This mechanism has been demonstrated to involve the ability of DJ-1-expressing astrocytes to protect against oxidative stress by reducing the amount of neuronal thiol oxidation [26]. Another study showed that DJ-1 has a role in the maintenance of mitochondria within astrocytes themselves [27]. Knockdown of *Park7* reduced astrocytic mitochondrial motility in the same manner as rotenone treatment. Furthermore, a decrease in astrocyte mitochondrial membrane potential caused by rotenone treatment was exacerbated by *Park7* knockdown. Mitochondrial fission was also shown to be reduced in the presence of *Park7* knockdown and rotenone treatment, although no changes were detected in mitochondrial fusion or respiration.

### α-Synuclein

One of the pathological hallmarks of PD is accumulation of α-synuclein-positive cytoplasmic inclusions in neurons. α-synuclein is encoded by the *SNCA* gene, and mutations as well as duplication or triplication of the gene have been shown to lead to development of the disease. Expression of *SNCA* in astrocytes is low, whereas it is abundantly expressed in neurons 28, 29. However, there is some evidence that the low levels in astrocytes may still have functional relevance. Astrocytes have an important role in fatty acid metabolism in the brain [30], and a study of primary cultures of mouse *Snca*-KO astrocytes showed that the incorporation and distribution of the fatty acids arachidonic acid (AA) and palmitic acid was disrupted [31]. Nevertheless, most studies point to a role for α-synuclein as an exogenous stimulator of astrocytes. In postmortem PD brains, α-synuclein-positive inclusions have been found in astrocytes as well as in neurons 32, 33, 34, raising the suggestion that α-synuclein secreted by neurons is taken up by astrocytes. Multiple studies have now revealed that astrocytes can take up α-synuclein 35, 36, 37, 38, and this has been shown to occur via a TLR4-independent endocytosis pathway 37, 38. Endocytosed α-synuclein has been shown to localise to the lysosome [36], suggesting that astrocytes have a role in its removal and degradation, potentially maintaining a healthy environment in which neurons can thrive.

High concentrations of extracellular α-synuclein have been shown to induce a TLR4-dependent inflammatory response in primary astrocyte cultures 37, 38, 39. There appears to be a concentration dependence to this effect, and this may be important in understanding the development of pathology in PD. When α-synuclein is secreted from neurons, astrocytes can endocytose and degrade it, although it may be the case that, if the concentration of α-synuclein in the extracellular space increases above a certain threshold, an inflammatory response is induced and pathology begins to develop. It is also possible that astrocytes endocytose increasing levels of extracellular α-synuclein when the latter is at a high concentration, leading to the accumulation and formation of α-synuclein inclusions in astrocytes, as seen in PD brains. This accumulation could then lead to the dysregulation of other astrocyte functions, such as glutamate uptake and blood–brain barrier integrity. The effect of α-synuclein accumulation in astrocytes has been demonstrated in a mouse model in which mutant *SNCA* was overexpressed under an astrocyte-specific promoter [40]. These mice developed a neurodegenerative movement disorder and developed astrogliosis before the onset of symptoms. The affected astrocytes exhibited decreased expression of the glutamate transporters Glast1 and Glt1. They also displayed abnormal localisation of the water channel Aquaporin-4 (AQP4), which is involved in blood–brain barrier function and water transport. Dopaminergic neuron degeneration was marked, with loss in the SNc (60.5%) and **ventral tegmental area** (VTA) (26.1%), mirroring the relative susceptibilities of the different regions, as seen in PD.

### iPLA_2_

*PLA2G6* encodes group VI Ca^2+^-independent phospholipase A_2_ (iPLA_2_), an enzyme that catalyses the release of fatty acids from phospholipids. Mutations in iPLA_2_ cause infantile neuroaxonal dystrophy and neurodegeneration with brain iron accumulation and have recently been shown to cause PD [41]. Inhibition of iPLA_2_ has been shown to inhibit the release of the fatty acid AA from phospholipids in astrocytes [42]. Additionally, *Pla2g6* knockdown and inactive mutants of *Pla2g6* have both been shown to result in a reduction in calcium responses (duration, amplitude, and capacitative calcium entry) in primary astrocyte cultures [43]. In WT cultures, it has been shown that the increase in duration of ATP-mediated calcium responses as a result of LPS stimulation involves the upregulation of iPLA_2_, suggesting a link with TLR4 signalling [44]. ATP-mediated calcium signalling is thought to be important for astrocyte-to-astrocyte communication [45] and, therefore, this process may be disrupted in patients with iPLA_2_ mutations.

### ATP13A2

*ATP13A2* encodes the lysosomal type 5 ATPase protein (ATP13A2), mutations of which cause an early-onset form of PD [46]. Disease mutations have been shown to impair lysosomal function and to reduce the integrity of the lysosomal membrane 47, 48. So far, only one study has investigated the role of ATP13A2 in astrocytes [49]. In this study, *Atp13a2*-KO astrocytes were shown to exhibit increased expression of proinflammatory cytokines, and decreased expression of anti-inflammatory cytokines and neurotrophic factors, such as GDNF, compared with WT cells. This was demonstrated to be mediated through the upregulation of the lysosomal protease Cathepsin B, which was subsequently released into the cytosol via damaged lysosomal membranes, resulting in the activation of the NLRP3 inflammasome. Conditioned medium from *Atp13a2*-KO astrocytes was found to be less neurotrophic than medium from WT astrocytes, and was less able to protect dopaminergic neurons from MPP+ toxicity. Interestingly, the authors also found that, in response to MPP+ treatment of WT astrocyte cultures, ATP13A2 expression was reduced, suggesting that MPP+ toxicity proceeds through a similar pathway.

### LRRK2

Leucine-rich repeat kinase 2 (LRRK2) is a large protein with dual kinase and GTPase activity that is encoded by the *LRRK2* gene 50, 51. Mutations in *LRRK2* are the most common genetic cause of PD, and result in a patient phenotype similar to the idiopathic disease [52]. LRRK2 is expressed in neurons, astrocytes, and microglia in the human brain 53, 54. It has been widely implicated in the autophagy-lysosome pathway in many models, including astrocytes 55, 56. During the initiation of autophagy, the LC3 protein is lipidated and trafficked to the membrane of autophagic vesicles. Inhibition of LRRK2 kinase activity has been shown to increase the lipidation of LC3 in primary mouse astrocytes [55]. This may be indicative of either induction of autophagosome formation or inhibition of autophagosome/autolysosome degradation. Furthermore, expression of GFP-tagged, PD-causing LRRK2 mutants (R1441C, Y1699C, and G2019S) in primary mouse astrocytes has been shown to result in an increase in lysosome size [56]. This effect was dependent on kinase activity, and was associated with a reduction in lysosomal pH. It was also shown that protein levels of ATP13A2 were increased in the presence of the G2019S mutation. It is unknown whether this upregulation is a direct result of the LRRK2 mutation or a compensatory effect.

### GCase

The *GBA* gene encodes β-glucocerebrosidase (GCase), a lysosomal enzyme that is involved in glycolipid metabolism. Heterozygous mutations are a risk factor for developing PD [57], whereas homozygous or compound heterozygous mutations lead to the development of Gaucher’s disease, a lysosomal storage disorder [58]. Astrocyte activation is a hallmark of certain neuronopathic lysosomal storage disorders and, in some cases, has been suggested to precede neuronal degeneration 59, 60. Conditional *Gba*-KO mice that lack *Gba* only in neurons and macroglia (i.e., astrocytes and oligodendrocytes) have been found to display an increase in expression of cathepsin lysosomal proteases in both astrocytes and neurons [61]. Primary *Gba*-KO astrocytes from another study demonstrated a reduced number of LC3-positive puncta, indicating further deficits in the autophagy pathway [62]. Additionally, this study found that mitochondria in *Gba*-KO neurons and astrocytes exhibited decreased mitochondrial resting membrane potential and increased mitochondrial fragmentation, and showed that the fragmentation of mitochondria was more severe in astrocytes than in neurons.

### PINK1

*PINK1* encodes PTEN-induced putative kinase 1 (PINK1), a protein widely shown to be involved in mitophagy, a process that selectively degrades damaged mitochondria [63]. *Pink1* expression in the brain has been shown to increase as embryonic development progresses, and has an important role in the development of astrocytes [64]. *Pink1*-KO mouse brains exhibit a reduction in the number of astrocytes compared with WT mice [64]. In postnatal mouse astrocyte cultures lacking *Pink1*, proliferation was markedly reduced [65]. Proliferation of astrocytes in the brain is controlled, at least in part, by EGFR signalling, and further investigation showed that PINK1 regulates EGFR protein levels via an AKT/p38-dependent pathway. In addition, the mitochondrial health of the cells was affected, resulting in a reduction in ATP production, which also contributed to their decreased proliferation. Most PD-related mutations in *PINK1* are loss-of-function mutations [66] and might be expected to result in a similar reduction in astrocyte proliferation capacity and overall number. This could have serious implications for neuroprotection and general brain health.

### Parkin

Parkin is another protein that has been widely implicated in mitophagy [63], and is encoded by the *PARK2* gene. Postmortem studies of brains from patients with PD and *PARK2* mutations have shown the accumulation of α-synuclein inclusions in astrocytes 67, 68. Similarly to *PINK1*, *PARK2* mutations tend to confer a loss of function, and Parkin has been shown to have a role in astrocyte proliferation, as demonstrated by the decreased proliferation of *Park2*-KO astrocytes 69, 70. *Park2*-KO astrocytes have also been found to have decreased neurotrophic capacity, mediated by a reduction in glutathione secretion 69, 70. Furthermore, one study found that, alongside other glial cells, but not neurons, *Park2*-KO astrocytes had increased levels of damaged mitochondria [71]. Parkin has also been shown to be upregulated in astrocytes, but not in neurons, as part of the unfolded protein response, demonstrating that Parkin may exhibit cell type-specific functions [72]. Additionally, Parkin has been shown to be involved in the astrocyte inflammatory response. Activation via IL-1β was shown to result in Parkin downregulation, whereas activation via TNF-α was shown to induce Parkin upregulation [73].

## Converging Pathways

**Figure 2:**
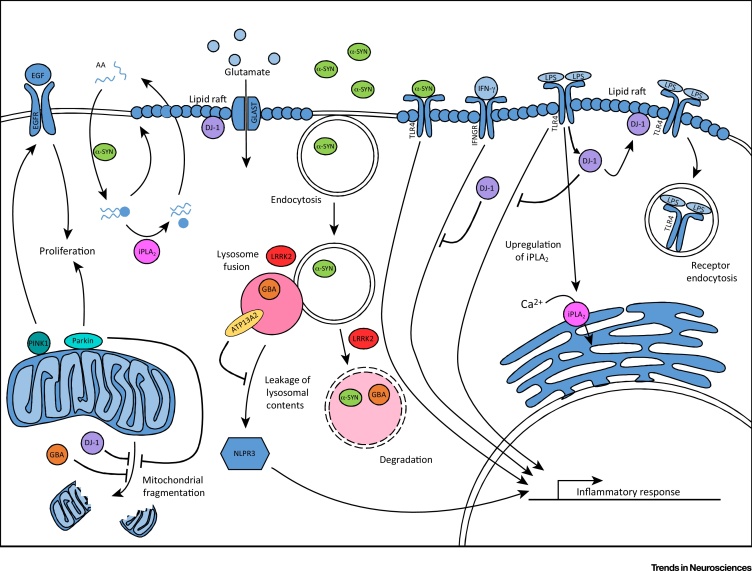
Parkinson’s Disease (PD)-Related Gene Pathways Are Implicated in Astrocytes. Parkin and PINK1 regulate proliferation, which, in the case of PINK1, occurs through the EGF signalling pathway. PINK1, Parkin, GBA, and DJ-1 have all been shown to have a role in the maintenance of healthy mitochondria. Astrocyte α-synuclein (α-SYN) regulates the uptake and distribution of arachidonic acid (AA), which is released from phospholipids by Group VI Ca^2+^-independent phospholipase A_2_ (iPLA_2_). DJ-1 regulates the stability of lipid rafts, therefore maintaining the glutamate transporter GLAST at the membrane. Additionally, DJ-1 is involved in the termination of TLR4 signalling via receptor endocytosis, and the inhibition of the IFN-γ inflammatory response. iPLA_2_ is upregulated in response to TLR4 signalling and increases the calcium load in the endoplasmic reticulum. Extracellular α-SYN can be endocytosed via a TLR4-independent process and degraded by the lysosome. At high concentrations, extracellular α-SYN activates TLR4 signalling that is not terminated by receptor endocytosis. GBA mutations can disrupt degradation of proteins via the autophagy pathway. Leucine-rich repeat kinase 2 (LRRK2) regulates fusion and/or degradation in the autophagy pathway and, alongside lysosomal type 5 ATPase (ATP13A2), may have a role in the control of lysosomal pH. ATP13A2 levels maintain the stability of the lysosome and prevent its contents from leaking into the cytosol, which can result in activation of the NLPR3 inflammasome. Abbreviation: LPS, lipopolysaccharide.

**Figure 3:**
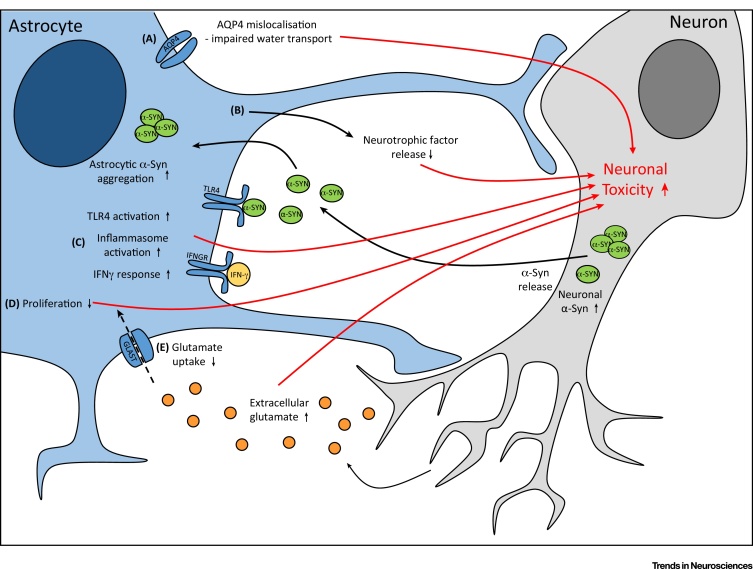
Dysfunctional Astrocytes Contribute to Neuronal Toxicity. Astrocyte dysfunction elicits neuronal toxicity via five main mechanisms. (A) Aquaporin-4 (AQP4) water channels are mislocalised away from the astrocyte end-feet, resulting in impaired water transport. (B) The neuroprotective capacity of astrocytes is reduced because of decreased neurotrophic factor release. (C) Inflammatory signalling via the TLR4, IFN-γ, and NLPR3 inflammasome pathways is increased. (D) Astrocyte proliferation is impaired, reducing the capacity of the cells to respond to an insult. (E) Glutamate uptake is reduced, potentially resulting in increased extracellular glutamate and, therefore, neuronal excitotoxicity. Abbreviation: α-SYN, α- synuclein.

The roles of each of the eight PD genes described above that have been studied to date converge on four main cellular functions: inflammatory response, lipid handling, mitochondrial health, and lysosomal function (Figure 2). Perturbation of these functions by mutations in PD genes results in astrocyte dysfunction, which has been shown in multiple cases to be detrimental to surrounding neurons (Figure 3).

### Inflammatory Response

As discussed above, multiple genes that have been implicated in PD have been demonstrated to have a role in the initiation and regulation of astrocyte activation in response to inflammatory stimuli. Not only have high levels of exogenous α-synuclein been shown to initiate a TLR4 signalling cascade 37, 38, 39, but there is also evidence that this signalling pathway is regulated by DJ-1 14, 20, 21 and has downstream effects on the expression of iPLA_2_ [44]. Furthermore, LRRK2 has been implicated in TLR4 signalling in microglial cells, although it is yet to be seen whether it has a similar role in astrocytes 74, 75. It has also been shown that DJ-1 regulates astrocyte activation by IFN-γ [22], Parkin expression is modified by IL-1β and TNF-α stimulation [73], and functional ATP13A2 expression at the lysosome prevents a cascade of events that results in activation of the NLRP3 inflammasome [49]. Disruption of these inflammatory signalling pathways has been shown to result in changes to essential astrocyte functions, including glutamate transport 15, 40, water transport [40], and neurotrophic capacity 22, 23, 24, 25, 49, 69. All of these functions are important for neuronal health and it has been demonstrated that these changes in astrocytes result in the degeneration of neighbouring neurons.

### Lipid Handling

As well as responding to inflammatory cues in the brain, astrocytes have a role in the metabolism of lipids and the release of fatty acids. This process has been shown to be disrupted in PD gene-KO models, especially in the case of the fatty acid, AA. *Pla2g6*-KO astrocytes display a diminished ability to release AA from phospholipids [42], whereas, in *Snca*-KO astrocytes, it is the uptake and distribution of AA that are disrupted [31]. Additionally, DJ-1 has been shown to have a role in the stability of lipid rafts 14, 15, which have been shown to be enriched in AA in other cellular models [76]. Furthermore, AA is an important modulator in the brain and is involved in astrocyte control of vasodilation and vasoconstriction 77, 78. It has also been shown to have a role in the modulation of astrocyte calcium oscillations and the TLR4-mediated inflammatory response 79, 80.

### Mitochondrial and Lysosomal Function

Further to the roles that PD-related genes have in astrocyte inflammatory response and fatty acid metabolism, we have discussed their role in global cellular pathways, such as mitochondrial function and lysosome biology, in the context of astrocytes. Dysfunction of both of these cellular processes been implicated in both familial and idiopathic PD 81, 82. In the cases of DJ-1, Gcase, and Parkin, KO models have shown disruption of mitochondrial function in astrocytes 27, 62, 71. In studies of Gcase and Parkin, a comparison was made with neurons and the disruption was found to be more pronounced in astrocytes. As yet, it is unclear whether mitochondrial damage is a cause or consequence of other cellular phenotypes, although it has been implicated both up- and downstream of inflammatory pathways in different models. One study found that mitochondrial damage occurs downstream of IL-1β and TNF-α expression [83], whereas another showed that it results in activation of the NLPR3 inflammasome [84].

With regards to lysosome biology, astrocyte uptake of α-synuclein from the extracellular space via endocytosis results in its degradation by lysosomes [36]; however, at high α-synuclein concentrations, this process may become overloaded, resulting in the accumulation of α-synuclein in the cytosol. This accumulation may lead to the aggregation of α-synuclein and formation of Lewy bodies, the key pathological hallmark of PD. Furthermore, perturbations in GCase, Atp13a2, and Lrrk2 function have all been shown to be detrimental to lysosome function in astrocytes 49, 56, 61. These effects have not been directly compared in different cell types and, thus, it remains to be seen whether they are exacerbated in, or specific to, astrocytes (see Outstanding Questions). Nevertheless, these changes in essential cellular functions may lead to a reduced ability of astrocytes to maintain a healthy environment for their neighbouring neurons to thrive in. To further support this hypothesis, there is evidence that astrocyte proliferation during development and in response to insult may be compromised by loss-of-function mutations in *PINK1* and *PARK2* 64, 65, 69, 70. This results in a decrease in the number of astrocytes present and, as such, each astrocyte has to work harder to maintain a healthy environment, and may fall short of this aim.

## Concluding Remarks

Although the number of studies that have been conducted to investigate the contribution of astrocytes to the pathogenesis of PD is few compared with those investigating neuronal function, a picture of astrocyte involvement in the disease is beginning to emerge. Here, we have reviewed the literature supporting the concept that genes known to be causative in PD have important roles in astrocyte biology. We have drawn on this research to describe how the implicated pathways intersect in the involvement of astrocytes in PD. In the cases of DJ-1, α-synuclein, iPLA_2_, ATP13A2, PINK1, and Parkin, there is now clear evidence that they are involved in astrocyte-specific functions, including inflammatory responses, glutamate transport, and neurotrophic capacity. In the cases of LRRK2 and GCase, no studies have yet investigated their role in astrocyte-specific functions, but they have been shown to have a role in the general physiology of astrocytes. Clearly, a wealth of research points to a role for many of the PD genes in neurons, although there is now also substantial evidence to suggest that non-cell-autonomous processes contribute to development of disease pathology. We believe that further investigation of the role of astrocytes in PD will be paramount to furthering our understanding of the disease (Box 1 and Outstanding Questions), and may have important implications for the development of new treatments (Box 2).

Outstanding Questions Are astrocytes key players in the development of α-synuclein inclusions in neurons?

Can healthy astrocytes become overloaded by demand for degradation of high concentrations of extracellular α-synuclein?

Do dysfunctional astrocytes exhibit impaired α-synuclein degradation?

Does astrocyte dysfunction initiate, or merely exacerbate, PD pathology?

Are lysosomal and mitochondrial dysfunction in astrocytes initiating factors or downstream consequences in PD?

Are astrocyte-derived neurotrophic molecules capable of slowing PD progression?

Do cell therapy approaches to treating PD need to include the transplantation of healthy astrocytes alongside neurons to succeed?

Box 1 **Modelling the Contribution of Astrocytes to PD**

In this article, we have discussed data produced from various models, including studies conducted in whole animals and in primary culture. In two animal models, cell type-specific KO (*Gba*) or overexpression (*SNCA*) was conducted to dissect the contribution of individual cell types to the pathogenesis of disease. The overexpression of mutant *SNCA* under an astrocyte-specific promoter resulted in the degeneration of dopaminergic neurons in the SNc and VTA as well as the onset of a movement disorder. It will be valuable to develop similar astrocyte-specific overexpression and KO models for other genes that have been shown to cause PD, to determine whether they also develop such phenotypes.

Likely due to the lack of availability of living human brain tissue for the derivation of primary cultures, the only human data available to cover in this review refer to postmortem pathology. All mechanistic insights into the role of PD-causative genes have been generated using animal models. However, it has been found that there is some variability in the expression levels of PD-related genes between human and mouse astrocytes and neurons (Figure 1, main text). This could prove especially pertinent in cases where the relative expression levels between these cell types is altered, and poses the question of how suitable animal models are for understanding the contribution of different cell types to the pathogenesis of human diseases, such as PD. The recent development of human **induced pluripotent stem cell** (iPSC)-derived astrocytes may go some way to enable the research community to answer this question [85].

Alt-text: Box 1

Box 2 **Therapeutic Implications**

Current treatments for PD act to alleviate the symptoms of the disease and do not alter disease progression. Deep brain stimulation (DBS) is one such treatment that can be used to successfully alleviate the motor symptoms of PD, and it has been demonstrated that its mechanism of action may include the activation of astrocytes [86]. Furthermore, clinical trials have been conducted to determine whether the delivery of GDNF, to promote dopaminergic neuron survival in the SNc, would be a suitable treatment for the disease. Results so far have been mixed, with some studies showing improvements in patients compared with controls and others showing no difference 87, 88, 89, 90. It will be important to determine whether small molecules, such as GDNF, can be successfully used to mimic the presence of healthy astrocytes, or whether treatment of astrocytic dysfunction or replacement of the astrocytes themselves will be necessary.

Previous studies have shown some benefit of transplanting human foetal midbrain tissue into the brains of patients [91]. Due to practical and ethical implications of conducting such studies on a larger scale, grafts of stem cell-derived dopaminergic progenitor cells are now being developed [92]. If astrocyte dysfunction is key to the development of PD pathology, merely replacing the lost dopaminergic neurons alone may not be successful. Therefore, it will be important to ensure that stem cell-derived grafts have the capacity to produce astrocytes alongside dopaminergic neurons to promote their ongoing survival.

There are promising data that show a reduced risk of developing PD in humans who have taken nonsteroidal anti-inflammatory drugs [93], and clinical trials of drugs that target inflammation in the brain are ongoing (NCT02787590). Other potential avenues for treatment may include the modulation of phospholipid and fatty acid processing in astrocytes, potentially via small molecules that chaperone or activate GCase [94]; regulation of glutamate in the extracellular space via modulation of glutamate transporters [95]; or enhancing the capacity of astrocytes to attenuate oxidative stress 96, 97. Furthermore, the recent generation of viral vectors to specifically target astrocytes may enable the direct modulation of astrocyte cellular function with minimal effects on other neighbouring cell types [98].

Alt-text: Box 2

## Glossary

**Dopaminergic neuron**: a neuronal subtype that synthesises and releases dopamine.
**Induced pluripotent stem cell (iPSC)**: a type of stem cell that can be derived from adult tissue, such as skin or blood cells. They retain the genetic information from the donor and can be differentiated into any cell type in the body.
**Substantia nigra pars compacta (SNc)**: a pigmented region of the midbrain where a subtype of dopaminergic neurons that selectively degenerate in PD resides.
**Ventral tegmental area (VTA)**: an area of the midbrain, rich in dopaminergic and serotonergic neurons. This region is more resistant to degeneration in PD than the cells in the SNc.
